# Individual Goffin´s cockatoos (*Cacatua goffiniana*) show flexible targeted helping in a tool transfer task

**DOI:** 10.1371/journal.pone.0253416

**Published:** 2021-06-29

**Authors:** I. B. Laumer, J. J. M. Massen, P. M. Boehm, A. Boehm, A. Geisler, A. M. I. Auersperg

**Affiliations:** 1 Department of Cognitive Biology, University of Vienna, Vienna, Austria; 2 Department of Anthropology, University of California, Los Angeles, Los Angeles, California, United States of America; 3 Animal Behaviour and Cognition, Department of Biology, Utrecht University, Utrecht, the Netherlands; 4 Messerli Research Institute, University of Veterinary Medicine, Vienna, Austria; Oregon Health and Science University, UNITED STATES

## Abstract

Flexible targeted helping is considered an advanced form of prosocial behavior in hominoids, as it requires the actor to assess *different* situations that a conspecific may be in, and to subsequently flexibly satisfy different needs of that partner depending on the nature of those situations. So far, apart from humans such behaviour has only been experimentally shown in chimpanzees and in Eurasian jays. Recent studies highlight the prosocial tendencies of several bird species, yet flexible targeted helping remained untested, largely due to methodological issues as such tasks are generally designed around tool-use, and very few bird species are capable of tool-use. Here, we tested Goffin’s cockatoos, which proved to be skilled tool innovators in captivity, in a tool transfer task in which an actor had access to four different objects/tools and a partner to one of two different apparatuses that each required one of these tools to retrieve a reward. As expected from this species, we recorded playful object transfers across all conditions. Yet, importantly and similar to apes, three out of eight birds transferred the *correct* tool more often in the test condition than in a condition that also featured an apparatus but no partner. Furthermore, one of these birds transferred that correct tool *first* more often before transferring any other object in the test condition than in the no-partner condition, while the other two cockatoos were marginally non-significantly more likely to do so. Additionally, there was no difference in the likelihood of the correct tool being transferred first for either of the two apparatuses, suggesting that these birds flexibly adjusted what to transfer based on their partner´s need. Future studies should focus on explanations for the intra-specific variation of this behaviour, and should test other parrots and other large-brained birds to see how this can be generalized across the class and to investigate the evolutionary history of this trait.

## Introduction

An intentional behavior benefiting another individual at low or no cost to the actor, in other words prosociality [1], emerges early in our development, long before socialization plays a major role, suggesting that humans may be pre-disposed to act prosocially [e.g. 2]. Current research suggests that at least some social animals may also act so that another individual profits by that action. To test basic prosocial behavior in non-humans, there are presently two main approaches; prosocial choice tasks in which an animal has a selection of choices of which only one benefits a partner, and instrumental helping tasks in which a token or tool is out of reach for a partner, yet can be transferred by the animal without self-benefit [reviewed in 3]. Studies on our closest relatives revealed mixed findings across tasks [4]. Some apes helped a human partner or conspecifics to reach for an object/tool [e.g. 5, 6] and chimpanzees, specifically, tended to aid a partner by opening a door to allow food access [7], or press a button to release juice for a conspecific [8], but they seemed to lack prosocial tendencies in prosocial choice tasks [e.g. 9, 10, but see 8, 11]. By now, many primate species have been tested and some differences in prosocial tendencies have been attributed to differences in the socio-ecology of those primate species [e.g. 12].

In order to better understand the evolutionary origins of prosocial behavior, however, recent studies additionally have started to focus on a variety of distantly related, non-primate species [13]. For example, it was found that wolves are prosocial, whereas, in the same task, dogs are not [14, but see 15]. In contrast, both meerkats and bottlenose dolphins failed to show any prosocial preferences in a prosocial choice task and an instrumental helping task respectively [16, 17]. Outside of mammals, most research effort has focused on large brained birds. As with the first primate studies the findings have been mixed. In corvids, for example, common ravens failed to show prosocial tendencies in a token exchange, as well as in a prosocial choice task [18, 19]. In contrast, Pinyon jays did show clear prosocial preferences in a choice task [20], and azure-winged magpies tested both in a group service paradigm and a food-sharing paradigm revealed high levels of proactive prosociality [21, 22]. As with primates, a recent comparative study among eight different corvid species revealed that variation in prosocial tendencies can be explained by species’ socio-ecology, and both cooperatively breeding and colonial breeding corvid species show more prosociality than other corvid species [23].

Among parrots, kea initially showed a preference for the option that benefited their partner in a choice task, but kept that preference in the control conditions, most likely due to a stronger food-association with that option [24]. Two grey parrots were also tested in a choice task that included a partner benefit, yet the dominant bird began by being prosocial but desisted after the subordinate failed to reciprocate [25, 26]. The ability to act prosocially was further tested in grey parrots in a token choice paradigm [27] and in grey parrots and blue-headed macaws in a classic instrumental helping/token transfer task [28]. In the former the parrots did only behave prosocially when the roles of being actor and recipient were altered but showed no increased prosocial choices when the birds stayed merely in the actor role, however, the behavior in the control conditions revealed that birds did not fully understand the task´s contingencies [27]. In the latter, however, a few Grey parrots, but not the blue-headed macaws, did also show spontaneous prosocial behavior in the test compared to the control conditions [28], suggesting that this trait may have also convergently evolved in, at least some, parrots.

An important and more advanced form of prosocial behavior that is often linked to empathy is flexible targeted helping, in which an animal acts specifically upon a partner’s need [5]. Anecdotal evidence for helping exists in large brained and highly social species, such as cetaceans biting through harpoon lines and elephants supporting weak conspecifics [e.g. 29, 30], and empirical evidence of what could also be described as ‘instrumental helping’ further exists in the form of freeing a caged conspecific in rats and common marmosets [e.g. 31, 32]. Flexible targeted helping, however, requires the actor to assess *different* situations that a conspecific/partner may be in and to subsequently flexibly satisfy different needs of that partner depending on the nature of those situations [33]. It is thus considered an advanced variant of pro-social behavior. Flexible targeted helping in the context of courtship behavior has previously been assessed in azure-winged magpies and Eurasian jays using different food-sharing paradigms in which, respectively, the actor either has access to food and can decide to share based on the availability of food for the partner [e.g. 21], or where the actor has access to two kinds of food and shares that on which its partner was not previously satiated [34], suggesting desire-state attribution.

Alternatively, researchers have used tool transfer paradigms that require specific decisions on the part of the actor about which tool to transfer depending on the situation its partner is in. In order to transfer the appropriate token/tool to a conspecific, an animal needs to i) attribute more than one tool/token to specific and variable applications, ii) recognize which tool/token a partner needs in the present situation, iii) select the appropriate tool/token out of an array of several objects/tools and, most notably, iv) be prosocially motivated to act upon the other´s need. Chimpanzees were tested in a tool transfer task in which one or both apes had access to one of two apparatuses and one or both animals were supplied with either matching or non-matching tools. The apes did transfer tools upon the partner´s request but only rarely if the tool worked for their own apparatus [33]. In a follow-up study [35], only one of the chimpanzees had access to the tools, while the other only to the apparatus. Five non-functional distractor objects, that were not associated with food rewards, were added to the two previous tools. The apes transferred the functional tools above chance level and more so when they could see into their partner’s compartment [35], suggesting that they flexibly adjusted their helping behavior based on the needs of their partner. In a recent study that included a helping and cooperation task, chimpanzees did not transfer tools to their partner, except for a mother-daughter dyad, but female bonobos sometimes transferred tools to their same-sex partner even when they did not benefit themselves [36]. However, the bonobos transferred the correct tool randomly, contrasting previous findings in chimpanzees [35]. Furthermore, there is some suggestive evidence on targeted helping in orangutans using a token-transfer paradigm [37]. In contrast, bonobos did not show instrumental helping as they never transferred stones to a conspecific that had nuts but no tool to crack them, even though they did show clear prosocial preferences as they did transfer nuts to their conspecifics [38].

Evidence of flexible helping in birds in such a tool-transfer task is so far lacking, and even within the primate order, it seems in fact largely limited to chimpanzees. One obstacle in interpreting these findings, however, is that testing other species requires their ability to use different tools on different apparatuses as a precondition. Flexible tool use is extremely rare in animals [39], but not restricted to primates, and there are in fact a few non-primates that would qualify as suitable test candidates.

Goffin´s cockatoos (hereafter Goffins) are among the few large brained bird species that repeatably proved capable of flexibly using and choosing tools for different tasks [40, 41]. Although Goffins are not dependent on tool-obtained resources in the wild [42], they do innovate tool-related behaviors in various captive and possibly wild situations [43, 44]. Moreover, they are highly social, live in complex hierarchically structured groups [42] and show activities that are associated with prosocial tendencies such as food sharing with unrelated, same sex conspecifics (IL, AA; repeated personal observation in captivity, throughout 2011 to present) and tolerated theft (IL, AA; repeated personal observation in field and captivity, throughout 2011 to present), as well as cognitive abilities related to cooperation such as inequity aversion [45].

**Fig 1:**
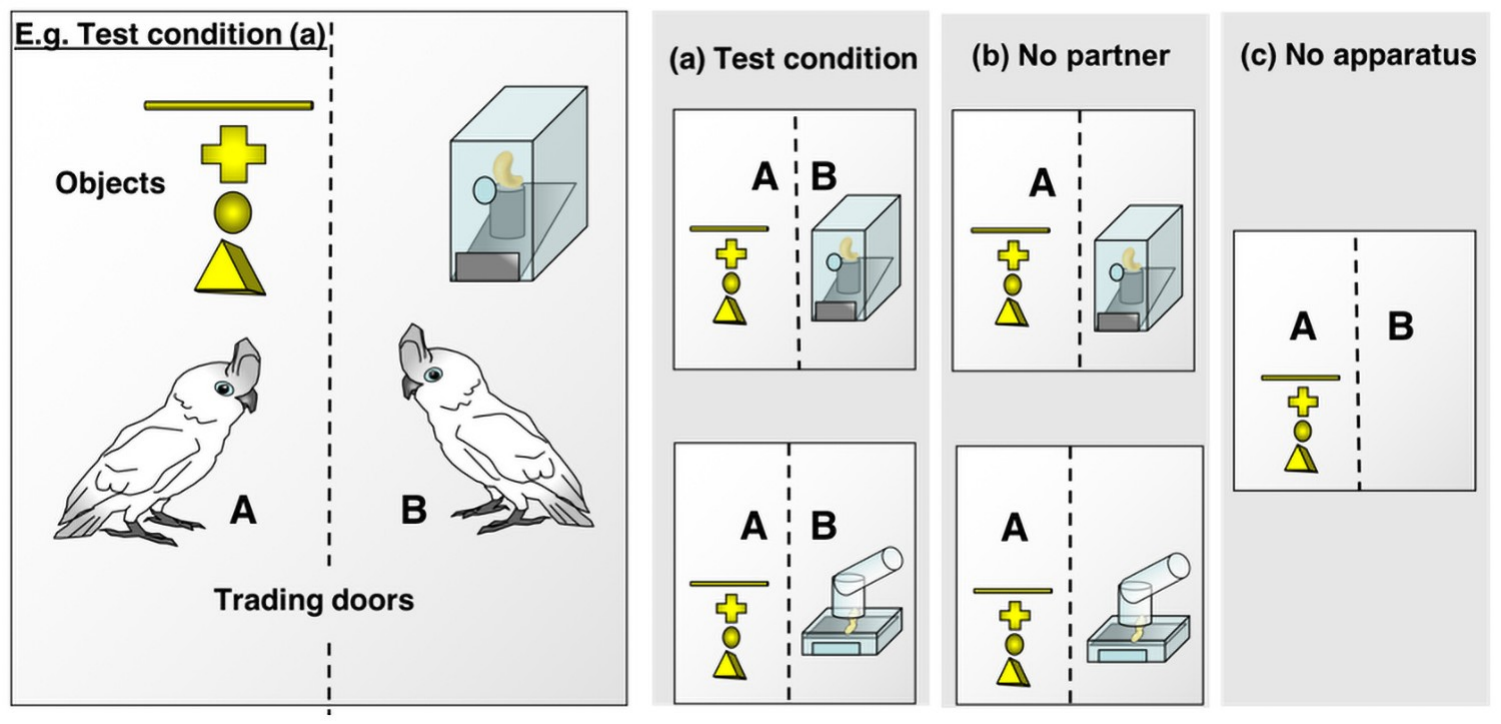
Overview of the three test conditions. Bird A is the acting parrot and bird B is the receiving parrot. a) test condition: both partner bird and stick- or ball apparatus present; b) no-partner control: the partner is missing but the apparatus in the partner´s compartment is present, and c) no-apparatus control: only the partner bird is present but no apparatus. In the test and non-social control condition, either the stick- or ball-apparatus was placed in the partner´s compartment.

For our design we decided to combine the setup of a classic instrumental helping /token transfer task with the tool-based setup used for studying flexible targeted helping. It thus included two distinct apparatuses (ball apparatus & stick apparatus), two experimental tools (ball & stick; each only functional with one of the two apparatus) and two distractor tools (cross and triangle), and all four objects were associated with food access from previous experience [40, 41]. Furthermore, we used a Plexiglas wall with small exchange windows to reduce the likelihood of accidental transfers. As in instrumental helping tasks, our setup included a ‘social’ and a ‘no-partner’ control. In the test condition the different tools were present in the actor´s compartment, whereas the partner´s compartment was supplied with either the baited stick- or the ball-apparatus only, and thus the question was whether the actor would transfer the correct tool to its conspecific such that this could operate the apparatus. The two control conditions were identical but in one the partner bird and in another the apparatus was missing (see Fig 1 & S1 Movie).

It is important to note here that aside from their flexible tool using abilities, Goffin’s cockatoos of all ages have an unusually strong drive for playful object combinations, including insertions of objects through cavities [46], and we thus expected a high rate of active object transfer through the window to occur playfully. Due to their play drive we would have expected them to fail the social and no-partner control in a classic instrumental helping/token transfer task, which was designed for setups that included a single functional token or tool. Nevertheless, our flexible design allowed us to specifically focus on the *order* and *types* of objects from our selection that would be preferred across the experimental conditions that we would present to them. While we expected playful transfers of non-particular object across all conditions, we would anticipate more of such transfers in the two partner conditions than in the no-partner condition if their play was largely socially motivated. If the birds paid attention to the presence of an apparatus they should show no preference for tool-type (stick/ball) objects in the condition in which the partner was lacking an apparatus (no-apparatus condition). If we did observe transfers across all conditions, transferring the *correct* tool proportionally more in the test condition over the condition in which no partner but an apparatus is present (no-partner condition) would support a targeted prosocial motivation account. Nevertheless, if the correct tool would be preferred in both apparatus conditions we could alternatively interpret the increased transfer of correct tools as an attempt to reach for the apparatus through the exchange window.

Evidence for flexible targeted helping in a tool transfer task in the Goffin would be a novel finding with regard to birds and would consequently suggest that the ability to specifically benefit another individual depending on the situation at hand developed convergently in these birds and those primates that also showed evidence of flexible targeted helping.

## Results

### Overall transfers

In overall transfers we did not find an obvious effect of condition on the total number of objects transferred (χ^2^ = 1.344, df = 2, P = 0.511). Moreover, neither session nor trial number revealed significant effects on overall transfers (S1 Table; S1 and S2 Figs in S1 File). 75% of test condition trials ended because the time elapsed and 62% of test condition trials ended without the actor transferring any tool.

### Targeted functional transfers and flexibility

**Fig 2:**
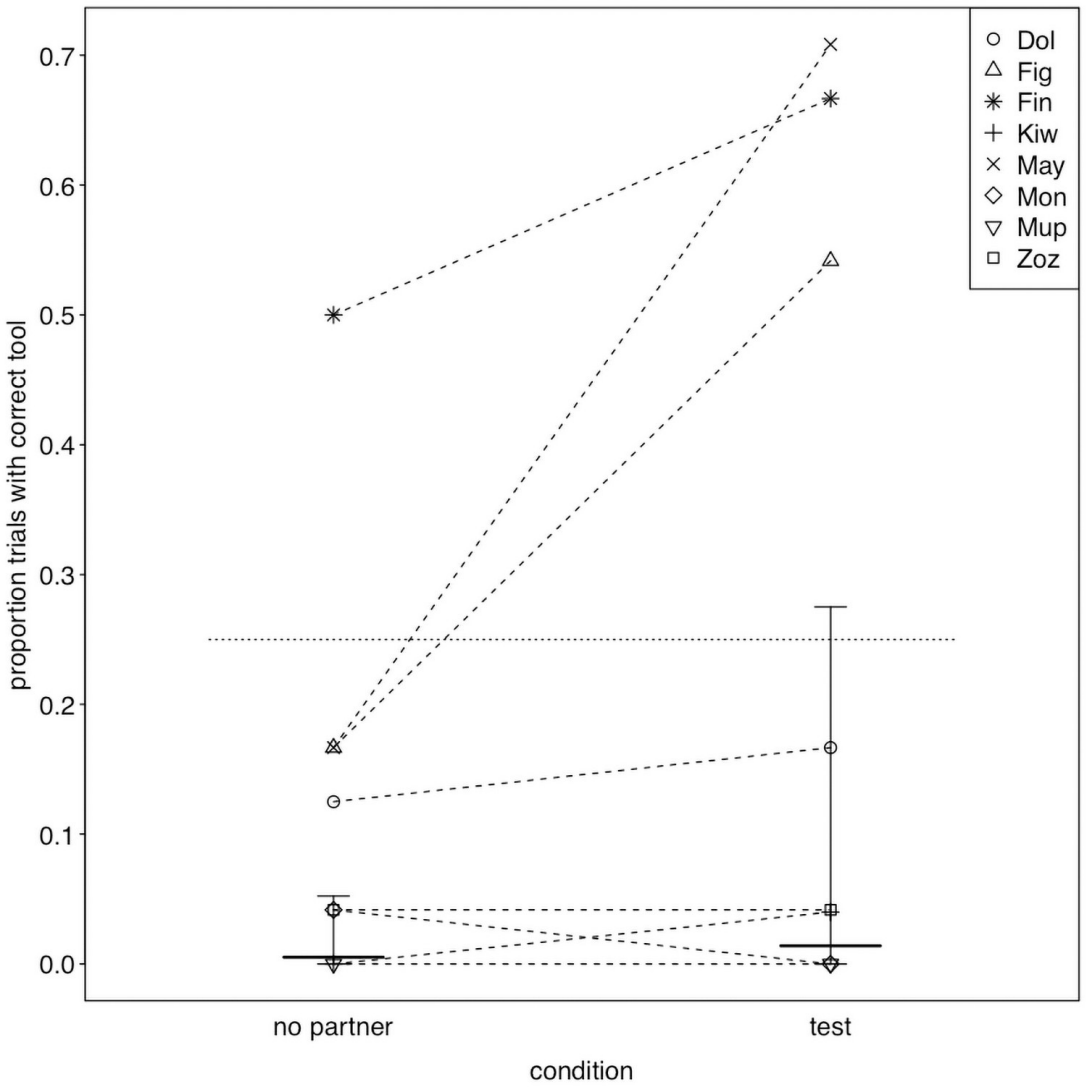
Proportion of trials in which the correct tool was transferred, separately for each actor and condition. Dotted lines connect observations from the same individual. The thick horizontal lines with error bars indicate the fitted model and its confidence limits (for session and trial number centered to a mean of zero). The horizontal dotted line depicts chance expectation.

**Fig 3:**
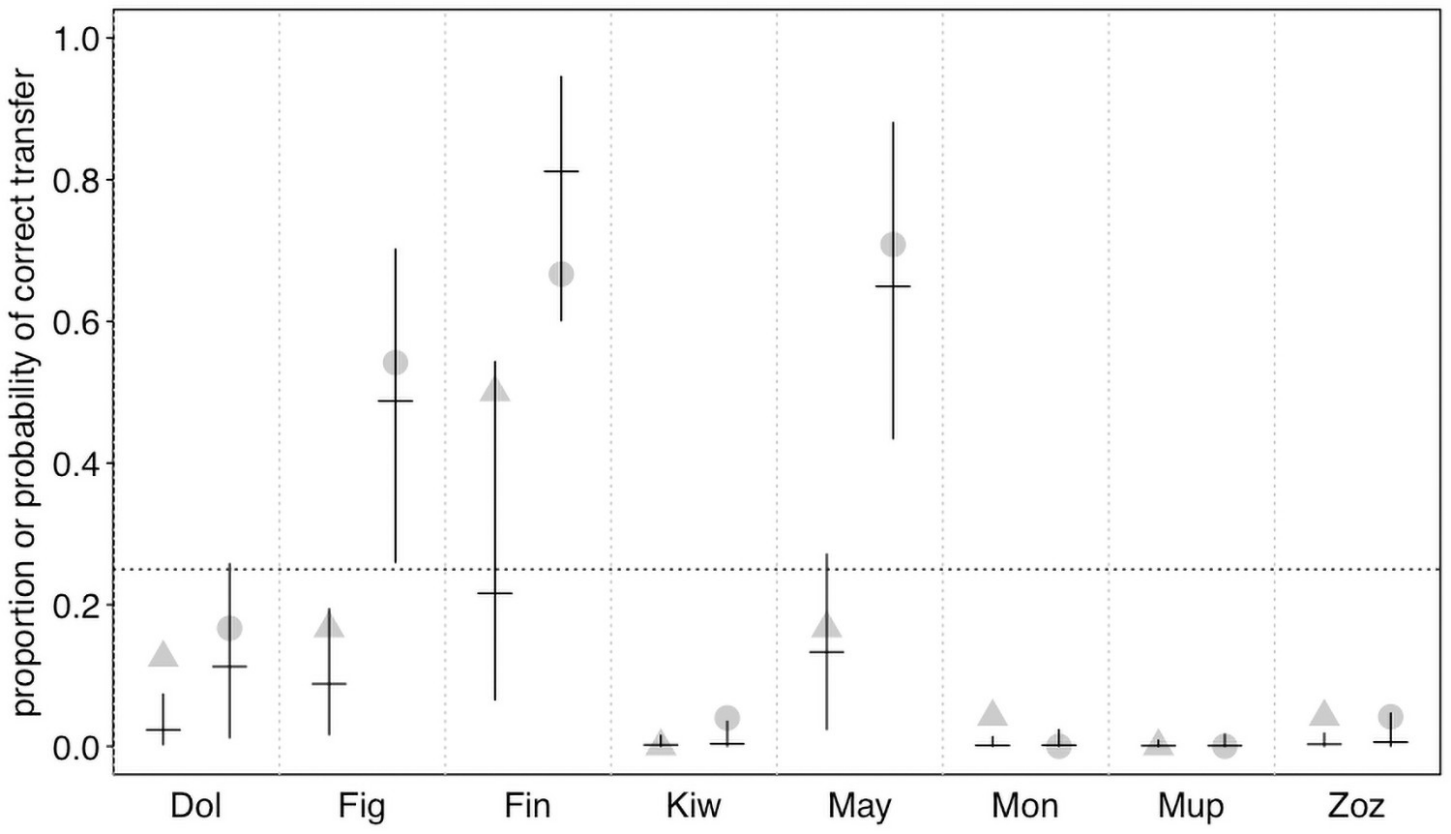
Proportion and probability of transferring the correct tool. For each individual, the no partner condition is depicted to the left (as triangles), and the test condition to the right (as circles). Dots show the individual performance and horizontal lines with error bars depict the fitted model and its confidence limits for the fixed effects intercept and condition effect as well as the random intercept of individual and the random slope of condition within individual. The horizontal dotted line depicts chance expectation. When the confidence interval for a given bird in a given condition does not comprise chance, this indicates that performance significantly deviate from chance.

**Fig 4:**
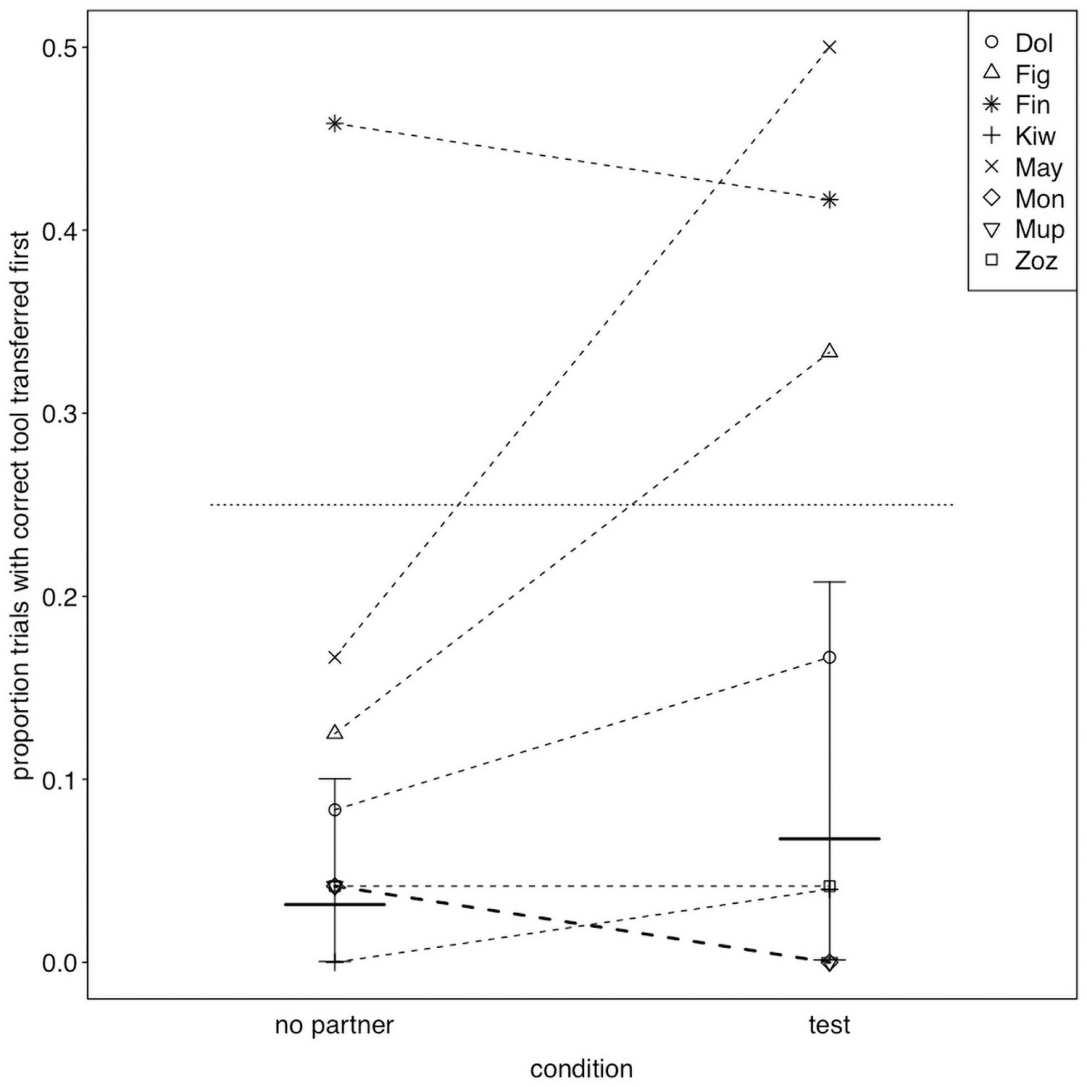
Proportion of trials in which the correct tool was transferred *first*, separately for each actor and condition. Dotted lines connect observations from the same individual (the thicker line indicated two individuals with tied observations). The thick horizontal lines with error bars indicate the fitted model and its confidence limits (for session and trial number centered to a mean of zero). The horizontal dotted line depicts chance expectation.

**Fig 5:**
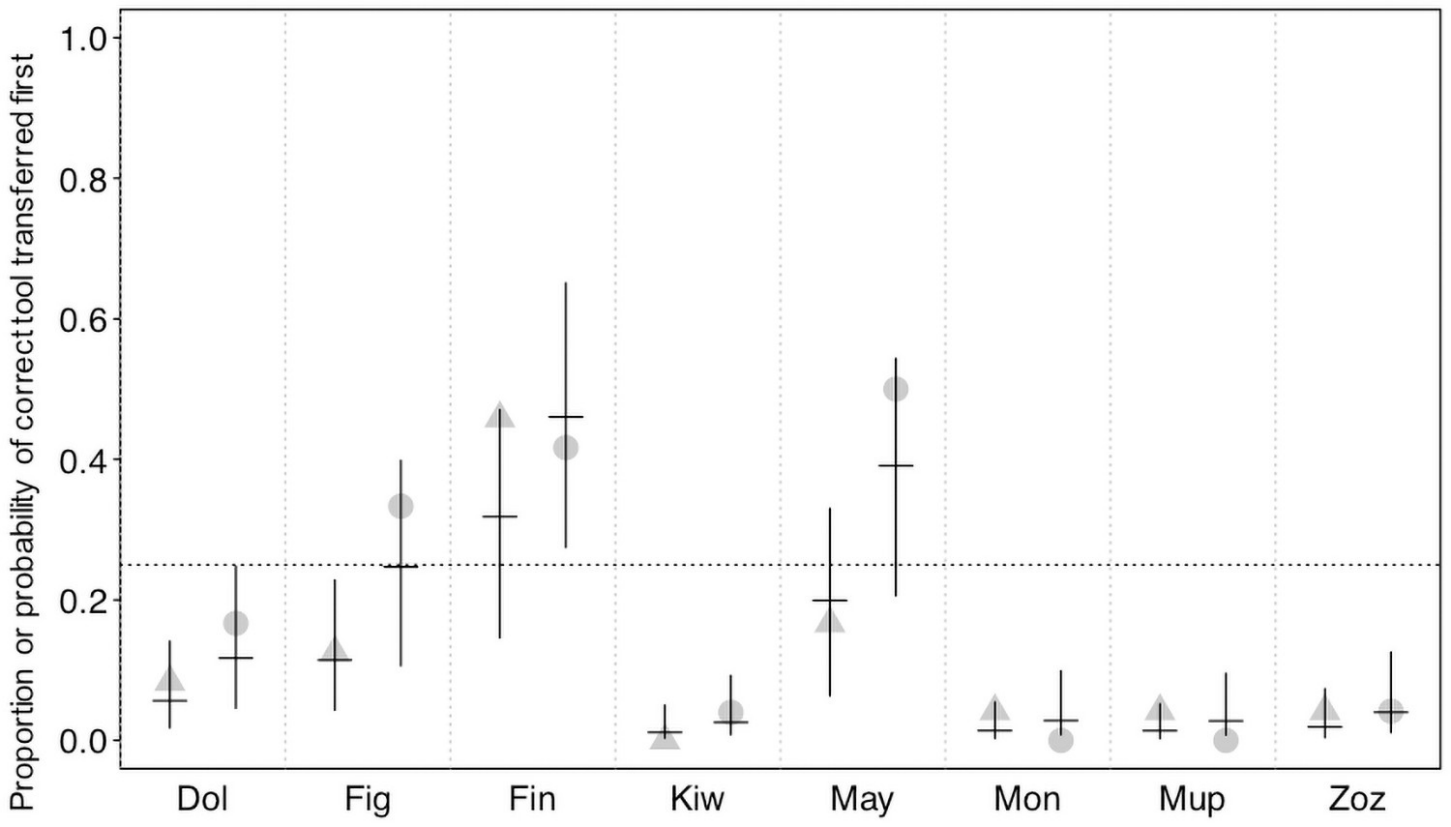
Proportion and probability of transferring the correct tool first. For each individual, the no partner condition is depicted to the left (as triangles), and the test condition to the right (as circles). Dots show the individual performance and horizontal lines with error bars depict the fitted model and its confidence limits for the fixed effects intercept and condition effect as well as the random intercept of individual and the random slope of condition within individual. The horizontal dotted line depicts chance expectation. When the confidence interval for a given bird in a given condition does not comprise chance, this indicates that performance significantly deviate from chance.

While the proportion of trials in which the correct tool was transferred (χ^2^ = 0.723, df = 1, P = 0.395; Fig 2, S3 Table in S1 File) and the proportion of trials in which the correct tool was transferred *first* (χ^2^ = 2.291, df = 2, P = 0.130; S4 Table in S1 File) did not significantly differ between conditions, we did find considerable differences between actors. This was also reflected by large estimated contributions of the random effects to the models (see S5 and S6 Tables in S1 File). In fact, three actors (Figaro, Fini and Mayday) did transfer the correct tool in most of the test trials and also significantly more frequently than in no-partner control trials (Fig 2, the confidence interval of one condition did not comprise the fitted value of the respective other, therefore they performed significantly differently in the two conditions, see Fig 3). In contrast, the remaining birds rarely transferred the correct tool and also showed little differences between the two conditions (Figs 2 & 3). We found similar results when considering whether the correct tool was transferred *first* (Fig 4, S4 Table in S1 File). Based on the confidence interval, one of the birds that transferred the correct tool significantly more often in the test than in the no-partner condition (Mayday) was also significantly more likely to transfer that correct tool *first* before transferring any other object in the test condition than in the no-partner condition (confidence interval of one condition did not comprise the fitted value of the respective other, see Fig 5), while the two other birds (Figaro and Fini) were marginally non-significantly more likely to transfer the correct tool *first* in the test condition than in the no-partner condition (see Fig 5). Note that these birds were also more likely to choose the correct tool to transfer (first) than would be predicted by chance (as there were 4 objects to choose from, chance level was set at 0.25, see Figs 3 & 5).

Overall, there was no significant effect of the apparatus on the probability to transfer the correct tool first (full null model comparison: χ^2^ = 0.902 df = 2, P = 0.637). Correspondingly, we did not find a significant interaction between apparatus and condition (χ^2^ = 0.000, df = 1, P = 0.991), and also no main effect of apparatus in a model from which we had removed the interaction (z = 0.745, P = 0.456; for full results of both models see S7 and S8 Tables in S1 File). This suggests that those individuals that did provide their partners the correct tools (*first*) in the test condition, could do so flexibly, and adjust their action to the specific need (i.e. tool) of their partner.

### Object preferences

When it comes to object preferences, we investigated how much time the birds spend manipulating each object in the condition in which no apparatus was present (as this may be associated with a certain tool), and found a slight trend regarding the birds’ preferences for manipulating a particular object (Friedman X^2^_r_ = 7.65, n = 8, df = 3, p = 0.054). Post-hoc contrasts (see S9 Table in S1 File) reveal, however, only one significant contrast and that is importantly between the two tools: A preference for the stick-tool over the ball-tool (note that this analysis only concerns the no apparatus condition), so they did not show a preference for either tools or distractor objects (proportion of time spend manipulating tools vs. distractor objects; Wilcoxon signed ranks test: W = 43, n = 8, p = 0.279).

### Seeking behavior

In 38.8% of the trials in which transfers occurred, we were able to observe ‘seeking behaviors’ of the partner bird that needed the tool (defined as: chewing the exchange window, reaching with the beak towards the correct tool behind the Plexiglass or putting the head through the exchange window), which could be considered as equivalent to what has been described as begging and attention getting behavior in primates. However, we found no significant difference between the no apparatus and the test condition with regard to the proportion of trials with seeking behaviour (χ^2^ = 0.221, df = 1, P = 0.638, S2 Fig, S10 and S11 Tables in S1 File). However, on an individual level the partners of the three birds, that did transfer the correct tool in most of the test trials and also significantly more frequently than in no-partner control trials, showed begging in more test trials than no-apparatus trials. The other five partner birds showed more begging in the no-apparatus trials than in the test trials (see S12 Table in S1 File).

## Discussion

All cockatoos transferred objects to the partner compartment and they often passed the objects back and forth and showed no difference between condition. This supports our assumption that many object transfers were playful. The play did not seem to be particularly socially motivated (we did not find significantly more transfers in the social conditions than in the no-partner condition). The cockatoos did also not show a general preference for the two tools over the other two objects in this condition.

Most notably however, when the partner and an apparatus was present (test condition) three out of eight birds transferred the correct tool more often than when the partner was absent (no-partner condition). Moreover, one of these birds was also more likely to transfer that correct tool *first* before transferring any other object in the test condition than in the no-partner condition, suggesting that at least these three birds were prosocially motivated and can flexibly adjust their tool transfers to the needs of their partners.

While it remains unclear why the effect we observed was limited to an individual level it is important to note that Goffins have complex social group structures [47] which may sometimes be unstable in captivity. Previous studies have highlighted the effect of relationship quality on prosociality [reviewed in 3; note that relationship quality also affected the results in various grey parrot studies, see 25–27]. Interestingly Goffin cockatoo Mayday, that was partnered up with Dolittle (her sibling), transferred the correct tool significantly more often in the test than in the no-partner condition and was the only bird that transferred the correct tool *first* before transferring any other object in the test condition than in the no-partner condition. However, note that transfers occurred rarely in the two other sibling dyads that participated in the test. While we tried to carefully select compatible dyads it was not possible to account for all interactions and shifts in social relationships that possibly occurred in the aviary during the experiment. Nevertheless, because three out of eight birds did show the effect it seems to be an existing but not a particularly widespread capacity within compatible dyads (note that dyads were chosen based on low aggression while maintaining a balance of male-female combinations and partnering birds of different rank).

Our results therefore suggest that, at least in in some birds, the tool transfers in conditions in which an apparatus was present were not mere attempts to reach the apparatus through the window with the appropriate tool, but required the combination of the presence of the partner and the apparatus, hence their actions seemed to be prosocial and flexible. Moreover, it is unlikely that the birds tried using their partner as a social tool [see 48], as food rewards were never shared with the actor.

Note here that the definition used to describe prosocial behavior in comparative psychology [1] does not imply that the actor has an empathic understanding of its partner’s need and/or desire but simply that it acts upon another individual’s benefit without the action being reciprocated. Prosocial behavior according to the previous definition seems to be more widespread than previously thought within mammals [e.g. dogs: 15; rats: 49] and seems to have evolved convergently in even more distantly related species such as birds [e.g. 21, 28]. Interestingly, even in primates, there seems to be no relation between prosocial behavior and phylogenetic closeness to humans [reviewed in 4]. Considering the evolution of prosociality within Aves, the combined facts that no other birds outside corvids and parrots have been tested on prosocial behavior, that previous studies of both groups produced rather mixed results [18–28, 50] and that corvids and parrots are divided by about 80 million years of evolution [e.g. 51], renders it unclear if the cognitive requirements for basic prosociality were present in the common ancestor of psittacopasserae. As an alternative it may have evolved convergently as a result of similar socio-ecological selection pressures [12, 23]. In order to unravel the evolution of prosocial behavior within Aves, further studies testing different avian species outside of these taxa are required.

As transfers of the correct tool in the aforementioned animals were preferred over transfers of other objects in the test condition, as preferences for the correct tool and for transferring the correct tool first occurred more often in the test condition than in the no-partner condition, and as the likelihood of the correct tool being transferred first did not differ for the two different apparatuses, we believe that their object preferences in this experiment fit present definitions of flexible targeted helping: they flexibly acted differently upon the benefit of a partner depending on the situation that their partner is presently in [e.g. 5; note again that a ‘correct tool’ does not exist in the no-apparatus condition]. Flexible targeted helping in nonhumans has been shown only in Eurasian jays [in a food sharing paradigm, 34] and in chimpanzees, but so far in none of the other nonhuman primates [e.g. 33, but see 36]. Our results thus suggest that such advanced forms of prosocial behavior are not necessarily specific to us and other hominids. In contrast, it may be present in distantly related, yet large brained animals which live in complex and hierarchically structured social groups.

Both chimpanzees and Goffins have the capacity to use tools flexibly [e.g. 33, 41, 52]. Interestingly however, non-habitually tool-using bonobos did not show any helping in a similar study design [38]. Whereas the Goffin does not depend on, nor is highly specialized on tool use, it is, like the chimpanzee, an extreme extractive forager with the capacity of innovate tool use in captivity [43], and we do not exclude the possibility of tool innovations spreading in the wild [44]. Nevertheless, we do not believe that the capacity for flexible targeted helping outside the limits of this particular setup is linked to tool use per se and it will thus be necessary to design less exclusive setups to study non-tool-using animals (possibly a setup including a token transfer and a matching-to-sample task). Interestingly, studies on desire state attribution in jays [e.g. 53] suggest that at least this corvid can flexibly adjust its behavior based on their partner’s specific need, albeit not in a truly prosocial context, but rather as part of courtship behavior.

Overall, the performance of the Goffins’ could be compared to other large brained species in setups on basic prosociality and to chimpanzees in setups on targeted helping, however as mentioned, on an individual level [e.g. 20–22, 28, 33, 35–37, 54]. Interestingly chimpanzees rarely helped their partner unless the action was directly requested [33]. Transfers were mostly initiated by a gesture of the partner. Due to morphological reasons, begging/seeking behavior towards an object in birds is harder to interpret than in apes. Nevertheless, we identified three potential forms of seeking behavior, such as pushing with the beak towards the tool behind the Plexiglas window or reaching with the head through the exchange window. In the Goffins, potential seeking behavior of the partner was equally likely in both partner conditions suggesting that the presence of an apparatus was irrelevant for it. A reason for this may be that all objects presented have previously been associated to food access in previous studies [40, 41] and/or as the animals’ extreme drive for playful object interaction [46]. Interestingly, on an individual level the partners of the three birds, that did transfer the correct tool in most of the test trials and also significantly more frequently than in no-partner control trials, showed begging in more test trials than no-apparatus trials, in contrast to the other five partners. Notably, in grey parrots and ravens the attention-getting behavior of the partners, such as vocalizations, reaching for tokens and creating sound by pecking at objects, did also not differ between partner conditions in a token exchange task [19, 28] while it did for jackdaws in a prosocial choice task [50] and azure-winged magpies in a food-sharing paradigm [22]. Tool transfers as a response to this seeking behavior or as a response triggered simply by the presence of one vs the other apparatus could be explained as a simple reaction to a cue, and thus by zero-order intentionality only [55]. Yet understanding which of the four tools / tokens the partner requires (as shown by transferring the correct tool *first* before transferring any other object in the test condition than in the no-partner condition (see Fig 5)) might suggest second-order intentionality, and possibly some sort of mental state attribution [55].

Taken together, our findings suggest that some form of prosocial behavior, one involving targeted helping according to the individual needs of a conspecific are expressed in the Goffin’s cockatoo on an individual level in a tool transfer context. Consequently, these data would suggest that this ability has evolved convergently at least twice in Aves. Additional comparisons with different bird species are required in order to determine the socio-ecological selection pressures that may have led to the evolution of this behavior specifically. Similarly, additional study designs that show targeted helping without the use of tools/tokens are needed to test whether targeted helping evolved as a by-product of instrumental object use in social animals.

## Methods

### Participants

Eight adult Goffin cockatoos (*Cacatua goffiniana*) were tested (see S13 Table in S1 File). The birds were housed at the Goffin Lab in Austria in a large group aviary (ca. 200 m^2^ ground space, up to 6m high) with in- and outdoor area. All parrots received water and food at libitum (fresh and dried fruits, vegetables, minerals, seeds, scrambled eggs) and participated in the experiments on a voluntary basis. During testing the cockatoos were separated from the group by asking them to enter the adjacent test compartment. Visual access to the group was blocked by closing the wooden sliding door between the test compartment and the aviary. As all experiments were appetitive, non-invasive and based exclusively on behavioural tests, they are not classified as animal experiments under the Austrian Animal Experiments Act (§ 2 Animal Experiments Act 2012, Federal Law Gazette I No. 114/2012). All animals had CITES certificates and were registered at the district’s administrative animal welfare bureau (Bezirkshauptmannschaft St. Pölten Schmiedgasse 4–6, A-3100; St. Pölten, Austria). These housing conditions comply with the Austrian Federal Act on the Protection of Animals (Animal Protection Act–§ 24 Abs. 1Z1 and 2;§25Abs.3 –TSchG,BGBl.INr.118/2004Art.2). All cockatoos had previous experience with tool use tasks and were familiar with the apparatuses used in this study [38, 39]. There were no differences in familiarity between birds as all cockatoos were kept together in a group aviary. Dyads were chosen based on low aggression while maintaining a balance of male-female combinations and partnering birds of different rank. The test was conducted in two parts. In part A, four birds were actors and four birds were recipients, while in part B the roles were reversed and actors coupled up with a new partner, as to avoid reciprocity motivating prosocial choices.

### Training in preparation for the test

#### Phase 1

To confirm the cockatoos´ knowledge about the functionality of the tools, they were confronted with either the ball- or the stick-apparatus and had to choose the correct tool out of two options (ball or stick; see S3 Fig in S1 File). The bird had to wait on the back of a chair in front of the testing table until the apparatus and both tools were placed on the table by the experimenter. The order of the apparatuses and the position of the tools (on the right and left side of the apparatus) were semi-randomly mixed throughout the sessions. The experimenter made sure to let go of both tools at the same time in order to avoid enhancing one of the options. The bird was then allowed to leave its starting position. As soon as one option was chosen, the other tool was immediately removed. If the wrong tool was chosen the bird had to wait for 30 seconds in a cage next to the testing table. After that the cage was opened and the bird went back to its starting position. The next trial started immediately upon the bird settling on the chairback. Each training session consisted of 12 trials. The birds were tested until they reached the criterion of at least ten out of 12 trials correct in two consecutive sessions.

#### Phase 2

In order to give the birds the experience that the two yellow distractor tools star and triangle shaped tools made out of FIMO that were functional in a previous study by Habl & Auersperg ([40], see S4 Fig in S1 File) are non-functional for both the ball- and stick-apparatus (S3 Fig in S1 File), the cockatoos were confronted with one apparatus (ball- or stick-apparatus) and either one of the distractor tools or the correct tool. To ensure that the distractor tools did not fit into the ball- or stick apparatus we used slightly enlarged versions of the original Fimo tools. The cockatoos received a total of two sessions of 10 trials each in which they received in semi-randomized fashion: eight trials in which they were confronted with one of the distractor tools and either the ball- or stick-apparatus. After the duration of one minute had elapsed the distractor tool was replaced with the correct tool. Furthermore, the birds received eight trials with the ball or stick tool and the respective apparatus. In the remaining four reminder trials, birds were presented with the original apparatus of the study by Habl and colleagues [40] and the functional original FIMO tool (see S4 Fig in S1 File).

#### Phase 3

To confirm that the birds choose the correct tool out of four different options (stick, ball, triangle, star), they received an unlimited number of sessions of 12 trials each. They were confronted with either the stick- or ball-apparatus in a randomized fashion. In order to habituate the birds to the testing cage, the training already happened in the cage with the plexiglas wall removed. The procedure was very similar to that of phase one. The four tools were always laid out from left to right in front of the baited apparatus and before giving the bird the start signal, all the tools were touched simultaneously with both hands to exclude stimulus enhancement. In contrast to phase 1 though, if the birds made the wrong choice they were not put into the adjacent cage for a time-out but the tools and the apparatus were immediately removed and the bird sent back to its starting position. The training continued until the cockatoos reached the criterion of at least 10/12 correct trials per session within two consecutive sessions. After completing all training phases, the birds entered the test. Since a previous study [41] shows that the cockatoos can choose the functional tool across different experimental setups involving different reward qualities, two tools (stick and ball) and the respective apparatuses without further training (note that we used the same tools and apparatuses as in current study), we did not continue to train the birds during the test.

### Preparations prior to test trials and details of the testing procedure

To keep the motivation to participate high, both actor and partner got a piece of cashew upon entering the test room and after the session was finished before they were brought back to the adjacent group aviary. Each trial was filmed separately from two angles, one from above the cage and one from the side. The cameras were adjusted to film the entire actor´s compartment and the plexiglass wall and as much as possible of the partner´s side.

If the actor threw both the stick and ball tool out of the test compartment (the two distractor tools did not fit through the wire mesh), the experimenter paused the timer, stood up, took the actor bird out of the its compartment, laid out all four tools again and finally touched all of them simultaneously. The timer was resumed when the bird was put in again. This procedure was repeated a maximum of three times and by the fourth time the trial ended (however this did never occur; the cockatoos rarely threw tools out of the testing compartment).

### Test setup

The cockatoos were sitting next to each other in two adjacent compartments separated by a plexiglas wall with exchange windows (Fig 1; for details see S7 Fig in S1 File). The birds seemed to accept the transparent wall as a barrier as none of them showed persistent behavioral signs that could be interpreted as attempts to squeeze through the very small transfer windows and get to the other side. Four different tools (ball, stick and two distractor tools, that were associated with food in a previous study [40] but were non-functional in the present study; see chapter b, S8 Fig in S1 File) were placed in the actor´s compartment while, depending on the condition, none or one of the two apparatuses was placed in the recipient´s compartment. Each apparatus could only be operated with one specific tool (stick or ball) and was baited with a high-quality food reward (cashew). All birds were trained individually in three different phases before entering the test (see above).

### Test conditions

In the test condition, both the actor, the recipient, one of the two apparatuses and the four tools were present (see Fig 1a). Furthermore, the birds were tested in an otherwise identical no-partner control, in which the apparatus was present in the recipient´s compartment, but not the recipient (see Fig 1b) and a no-apparatus control, in which both birds were present, but no apparatus was placed in the recipient´s compartment (see Fig 1c). The controls were conducted in order to assess whether the presence or absence of a partner or apparatus has an influence on the number of tool transfers and whether the birds transferred a tool upon their partner’s need or for other reasons, such as bringing the tool closer to the baited apparatus or for social play. Each of the three conditions was tested for four sessions (each with 6 trials) in a semi-randomized fashion, so that each condition was tested a total of 24 trials per dyad. During a session of the test and no partner condition the apparatus was changed after the 3rd sessions, and the order of the two different apparatuses was counterbalanced over the sessions.

### Testing procedure

Before a trial, one of the two apparatuses (semi-randomized) was baited and fixed with two screws on the floor of the test compartment. The position of the apparatus in the two compartments was semi-randomized. At the beginning of each trial, both cameras were switched on and the tools were placed in the compartment opposite of the apparatus from left to right. The order of the tools was semi-randomly mixed across sessions. The stick tool was placed facing the apparatus in a 90° degree angle, touching the cage wall on the door side and the other tools were aligned with the free end of the stick. To avoid stimulus enhancement all tools were covered with both hands before closing the doors of the cage.

First the partner bird was put in his compartment and the doors closed. Then the actor was put in the corner of the actor´s compartment and with the closing of the cage door a timer of three minutes was started. During the entire trial duration of up to three minutes the experimenter was sitting on a chair in the corner of the test room, was not speaking and moving and wore mirrored sunglasses to avoid any cueing. The trial ended when either the partner bird successfully operated the apparatus or the time elapsed.

### Analysis

Trials were recorded using HD video cameras (JVC HD Everio Camcorder GZ-HM30 or Samsung Galaxy S mini) and coded in situ as well as from the videos. The videos were coded using the Behavioral Observation Research Interactive Software BORIS [56]. Interobserver reliability was tested in advance and in-between by each person coding three videos of each condition individually (ICC ≥ 0.998; p<0.001). Furthermore, 15% of all videos of birds that transferred in more than 10 trials were double-coded and an excellent agreement was found (ICC ≥ 0.920; p<0.001).

#### Overall transfers

To test whether the number of overall transfers differed between conditions we fitted a Generalized Linear Mixed Model [GLMM; 57] with Poisson error structure and log link function [58]. Into this we included condition as our key test predictor with fixed effects. To control for their effects, we included session number and trial number as further fixed effects. To account for the duration of trials varying among them we included trial duration divided by 180 (roughly the average trial duration) and then log-transformed as an offset term [58]. As random intercept effects we included the identity of the individual and also the ID of the session, nested within individual. This latter random intercept effect accounts for possible within individual session to session variation in the total number of transferred objects. To avoid an overconfident model, we included random slopes [59, 60] of condition (manually dummy coded and then centered), session and trial number within individual and of trial number within session ID and also parameters for the correlations between random intercepts and slopes. Such random slopes account for (and model) the possibility that, for instance, the effects of condition vary between individuals. The sample considered for this model comprised a total of 577 trials in 96 sessions conducted with 8 actors during which at least one object was transferred in 178 trials. The model was not over dispersed (dispersion parameter: 0.817).

#### Targeted functional transfers and flexibility

To test whether the probability of transferring the right tool, and transferring the right tool first, we fitted two GLMMs with binomial error structure and logit link function. With regard to the fixed and random effects included, these models were identical to model of overall transfers described above. However, this time we included only data from the test and the no-partner control condition because there is no distinction between correct and incorrect tools in the no-apparatus condition. Furthermore, from the model with transfers of the right tool first, we excluded the correlations among random intercepts and slopes, as several of the absolute correlation parameters were close to one which is indicative of them not being identifiable [61]. The sample for both models comprised a total of 385 trials, conducted with 8 actors in 64 sessions. The number of trials in which the correct tool was transferred was 77, and the number of trials in which the correct tool was transferred first was 59. In order to further explore the individual specific probabilities to transfer the correct tool and also to transfer it first, we derived fitted values per individual using Best Linear Unbiased Predictors [BLUPs; 57] and also bootstrapped their confidence limits.

We also tested whether the probability of transferring the correct tool first differed between the two apparatuses. For this comparison we used only data from the test condition and no partner condition, as there was no ‘correct’ tool in the no apparatus condition. To address this question, we fitted a GLMM with binomial error structure and logit link function. Into this we included as fixed effects apparatus, condition, and their two-way interaction as well as trial and session number. As random intercept effects we included the identity of the actor, and we also included random slopes of all fixed effects (including the interaction) within actor. We also included parameters for the correlations among the random intercept and the random slopes. As an overall test of the effect of apparatus we compared this full model with a null model lacking apparatus with its interaction and condition. We determined model stability and confidence intervals of model coefficients as for the other models. The sample analyzed for this model comprised a total of 384 trials (in 59 of which the correct tool was transferred first) conducted with eight actors.

#### Seeking behavior

To test the probability of seeking behaviour to occur we fitted a GLMM with binomial error structure and logit link function, which was identical to the other models with the exception that we did not include parameters for the correlations between random intercepts and slopes because these appeared to be mainly unidentifiable, as indicated by absolute correlation parameters close to one [61]. Furthermore, we this time included only trials from the test and the no-apparatus condition. The sample analyzed for this comprised a total of 384 trials, conducted with 8 actors in 64 sessions. As we did not notice any contact calls from the birds in the test cage during the experiment, vocalization was not included as potential seeking behavior.

#### General considerations

We fitted all models in R [version 3.6.3; 62] using the function glmer of the package lme4 [version 1.1–21; 63]. We determined model stability by dropping levels of random effects one at a time and comparing the estimates obtained for models fitted to those subsets with those obtained for the full data set. This revealed the models to be mostly of good stability (see S1 and S2 Tables in S1 File). We determined confidence intervals of model estimates and the fitted models by means of parametric bootstraps (N = 1,000 bootstraps) using the function bootMer of the package lme4. For bootstrapping BLUPs we set the argument use.u of the function bootMer to TRUE. We determined the significance of individual fixed effects by dropping them from the full model one at a time and comparing these models with the respective full model, utilizing likelihood ratio tests [60, 64]. In all models we z-transformed session and trial number to a mean of zero and a standard deviation of one to ease model convergence.

## Supporting information

S1 Movie Movie of the test conditions. (MP4)

S1 Data All the data supporting the findings of this study are provided as a source data file. (CSV)

S1 File (DOCX)
