# Correction: Individual Goffin´s cockatoos (*Cacatua goffiniana*) show flexible targeted helping in a tool transfer task

**DOI:** 10.1371/journal.pone.0316744

**Published:** 2024-12-30

**Authors:** I. B. Laumer, J. J. M. Massen, P. M. Boehm, A. Boehm, A. Geisler, A. M. I. Auersperg

There are errors in the Author Contributions. The correct contributions are:

**Conceptualization:** I. B. Laumer, A.M.I. Auersperg

**Formal analysis:** J.J.M. Massen

**Funding acquisition:** A.M.I. Auersperg

**Investigation:** P.M. Boehm, A. Boehm, A. Geisler

**Methodology:** I.B. Laumer, J.J.M. Massen, A.M.I. Auersperg

**Resources:** A.M.I. Auersperg

**Supervision:** I.B. Laumer

**Writing–original draft:** I.B. Laumer

**Writing–review & editing:** J.J.M. Massen, A.M.I. Auersperg

## References

[1] Laumer IB, Massen JJM, Boehm PM, Boehm A, Geisler A, Auersperg AMI (2021) Individual Goffin´s cockatoos (Cacatua goffiniana) show flexible targeted helping in a tool transfer task. PLoS ONE 16(6): e0253416. 10.1371/journal.pone.0253416 34185776 PMC8241052

