# Supplementary material for: Individual Goffin´s cockatoos (Cacatua goffiniana) show flexible targeted helping in a tool transfer task
Source: PLoS One. 2021 Jun 29;16(6):e0253416. doi: 10.1371/journal.pone.0253416 (PMC8241052; doi:10.1371/journal.pone.0253416)
Supplement: S1 File — (DOCX) [file pone.0253416.s003.docx]

**Supplementary Material**

**Title**: Goffin´s cockatoos show flexible targeted helping in a tool transfer task

**Authors**: Laumer, I.B., Massen J.J.M., Boehm P.M., Boehm A., Geisler A., Auersperg A.M.I

#### **Additional results**


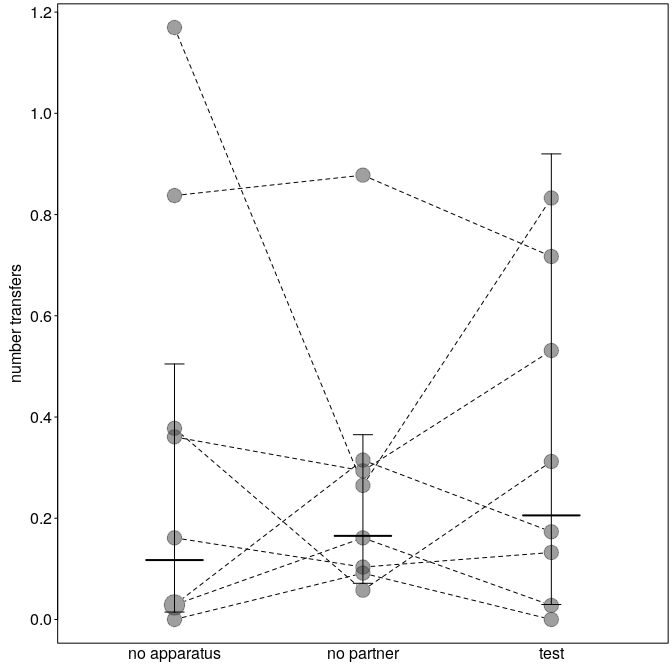


**S1 Figure** Number of transfers per actor and condition. Indicated are the back transformed logarithmic mean of the number of transfers after adding 1. Larger dots indicate tied observations (i.e., two individuals with the exact same number of transfers, and dotted lines connect observations from the same individual. The thick horizontal lines with error bars indicate the fitted model and its confidence limits (for session and trial number centered to a mean of zero and a trial duration of 180 seconds which roughly corresponds to the average trial duration).

**S1 Table** Results of the model with the number transfers as the response (estimate, together with standard error, confidence limits, test results, as well as minimum and maximum of estimates after deleting sessions and actors one at a time).

| term | Est. | SE | lower Cl | upper Cl | χ^2^ | df | P | min | max |
| --- | --- | --- | --- | --- | --- | --- | --- | --- | --- |
| intercept | -2.142 | 0.817 | -4.218 | -0.684 |  |  | ^(1)^ | -2.699 | -1.618 |
| condno partner^(2)^ | 0.341 | 0.731 | -1.024 | 2.231 | 1.344 | 2 | 0.511 | -0.151 | 0.702 |
| condtest | 0.560 | 0.490 | -0.431 | 1.729 |  |  |  | 0.033 | 0.767 |
| session nr.^(3)^ | -0.382 | 0.247 | -0.828 | 0.097 | 1.841 | 1 | 0.175 | -0.589 | -0.247 |
| trial nr.^(4)^ | -0.062 | 0.171 | -0.397 | 0.275 | 0.129 | 1 | 0.719 | -0.203 | 0.037 |

^(1)^ not indicated because of having a very limited interpretation

^(2)^ dummy coded with no apparatus being the reference category; the indicated significance test refers to the overall effect of the factor

^(3)^ z-transformed to a mean of zero and a standard deviation (sd) of one; mean and sd of the original variable were, 6.504 and 3.454, respectively

^(4)^ z-transformed to a mean of zero and a standard deviation (sd) of one; mean and sd of the original variable were, 3.496 and 1.711, respectively

**S2 Table** Results of the random effects part of the model with the number transfers as the response.

| grouping factor | effect^(1)^ | var2 | sd or corr |
| --- | --- | --- | --- |
| sessionID | intercept |  | 1.202 |
| sessionID | trial nr. |  | 0.477 |
| sessionID | intercept | trial nr. | 0.514 |
| Actor | intercept |  | 1.509 |
| Actor | cond.no.partner |  | 1.581 |
| Actor | cond.test |  | 0.213 |
| Actor | session nr. |  | 0.449 |
| Actor | trial nr. |  | 0.217 |
| Actor | intercept | cond.no.partner | -0.889 |
| Actor | intercept | cond.test | -0.080 |
| Actor | intercept | session nr. | -0.246 |
| Actor | intercept | trial nr. | -0.465 |
| Actor | cond.no.partner | cond.test | 0.527 |
| Actor | cond.no.partner | session nr. | 0.631 |
| Actor | cond.no.partner | trial nr. | 0.726 |
| Actor | cond.test | session nr. | 0.919 |
| Actor | cond.test | trial nr. | 0.717 |
| Actor | session nr. | trial nr. | 0.930 |

1. intercept denotes a random intercepts effect, all others a random slope (column 'var2' empty) or a correlation between a random intercepts and a ransom slopes effect (column 'var2' not empty)

**S3 Table** Results of the model with correct tool transferred (no/yes) as the response (estimate, together with standard error, confidence limits, test results, as well as minimum and maximum of estimates after deleting sessions and actors one at a time).

| term | Est. | SE | lower Cl | upper Cl | χ^2^ | df | P | min | max |
| --- | --- | --- | --- | --- | --- | --- | --- | --- | --- |
| intercept | -5.249 | 1.745 | -16.717 | -2.897 |  |  | ^(1)^ | -7.361 | -4.446 |
| condtest^(2)^ | 0.996 | 1.034 | -1.675 | 3.678 | 0.723 | 1 | 0.395 | 0.400 | 1.777 |
| session nr.^(3)^ | -0.806 | 0.494 | -2.250 | 0.282 | 2.090 | 1 | 0.148 | -1.198 | -0.551 |
| trial nr.^(4)^ | -1.724 | 1.037 | -5.719 | -0.233 | 5.526 | 1 | 0.019 | -2.617 | -1.105 |

^(1)^ not indicated because of having a very limited interpretation

^(2)^ dummy coded with no partner being the reference category

^(3)^ z-transformed to a mean of zero and a standard deviation (sd) of one; mean and sd of the original variable were, 6.538 and 3.412, respectively

^(4)^ z-transformed to a mean of zero and a standard deviation (sd) of one; mean and sd of the original variable were, 3.494 and 1.713, respectively

**S4 Table** Results of the model with correct tool transferred first (no/yes) as the response (estimate, together with standard error, confidence limits, test results, as well as minimum and maximum of estimates after deleting sessions and actors one at a time).

| term | Est. | SE | lower Cl | upper Cl | χ^2^ | df | P | min | max |
| --- | --- | --- | --- | --- | --- | --- | --- | --- | --- |
| intercept | -3.422 | 0.820 | -7.664 | -2.194 |  |  | ^(1)^ | -4.584 | -2.866 |
| condtest^(2)^ | 0.795 | 0.460 | -0.154 | 1.921 | 2.291 | 1 | 0.130 | 0.447 | 1.333 |
| session nr.^(3)^ | -0.472 | 0.235 | -1.037 | 0.033 | 3.400 | 1 | 0.065 | -0.646 | -0.325 |
| trial nr.^(4)^ | -0.428 | 0.258 | -0.987 | 0.093 | 2.905 | 1 | 0.088 | -0.591 | -0.318 |

^(1)^ not indicated because of having a very limited interpretation

^(2)^ dummy coded with no partner being the reference category

^(3)^ z-transformed to a mean of zero and a standard deviation (sd) of one; mean and sd of the original variable were, 6.538 and 3.412, respectively

^(4)^ z-transformed to a mean of zero and a standard deviation (sd) of one; mean and sd of the original variable were, 3.494 and 1.713, respectively

**S5 Table** Results of the random effects part of the model with correct tool transferred as the response.

| grouping factor | effect^(1)^ | var2 | sd or corr |
| --- | --- | --- | --- |
| sessionID | intercept |  | 2.788 |
| sessionID | trial nr. |  | 0.692 |
| sessionID | intercept | trial nr. | -1.000 |
| Actor | intercept |  | 1.878 |
| Actor | cond.test |  | 0.933 |
| Actor | session nr. |  | 0.475 |
| Actor | trial nr. |  | 0.340 |
| Actor | intercept | cond.test | 0.544 |
| Actor | intercept | session nr. | 0.564 |
| Actor | intercept | trial nr. | 0.877 |
| Actor | cond.test | session nr. | 1.000 |
| Actor | cond.test | trial nr. | 0.074 |
| Actor | session nr. | trial nr. | 0.098 |

^(1)^ intercept denotes a random intercepts effect, all others a random slope (column 'var2' empty) or a correlation between a random intercepts and a ransom slopes effect (column 'var2' not empty)

**S6 Table** Results of the random effects part of the model with correct tool transferred first as the response.

| grp | effect^(1)^ | sd |
| --- | --- | --- |
| sessionID | trial nr. | 1.181 |
| sessionID | intercept | 0.553 |
| Actor | trial nr. | 0.000 |
| Actor | session nr. | 0.218 |
| Actor | cond.test | 0.378 |
| Actor | intercept | 1.662 |

^(1)^ intercept denotes a random intercepts effect, all others a random slope

**S7 Table** Correct tool transfers first as a function of apparatus (full model; estimate, together with standard error, confidence limits, test results, as well as minimum and maximum of estimates after deleting sessions and actors one at a time).

| term | Estimate | SE | lower Cl | upper Cl | χ^2^ | df | P | min | max |
| --- | --- | --- | --- | --- | --- | --- | --- | --- | --- |
| (Intercept) | -4,174 | 1,735 | -11,903 | -1,946 |  |  | ^(1)^ | -5,438 | -3,236 |
| Condition ^(2)^ | 0,356 | 1,453 | -4,965 | 4,514 |  |  | ^(1)^ | -0,004 | 0,544 |
| Apparatus ^(3)^ | 1,114 | 1,621 | -2,131 | 7,747 |  |  | ^(1)^ | 0,248 | 2,666 |
| Session ^(4)^ | -0,515 | 0,323 | -1,505 | 0,154 | 2,629 | 1 | 0,105 | -0,665 | -0,377 |
| Trial ^(5)^ | -0,796 | 0,431 | -2,106 | -0,169 | 6,090 | 1 | 0,014 | -1,602 | -0,430 |
| Condition:Apparatus | -0,023 | 1,663 | -4,695 | 5,286 | 0,000 | 1 | 0,991 | -0,559 | 0,735 |

^(1)^ not indicated because of having a very limited interpretation

^(2)^ dummy coded with no partner being the reference category.

^(3)^ dummy coded with ball being the reference category.

^(4)^ z-transformed to a mean of zero and a standard deviation (sd) of one; mean and sd of the original variable were, 6.531 and 3.414, respectively.

^(5)^ z-transformed to a mean of zero and a standard deviation (sd) of one; mean and sd of the original variable were, 3.500 and 1.710, respectively.

**S8 Table** Correct tool transfers first as a function of apparatus (reduced model lacking the interaction; estimate, together with standard error, confidence limits and, test results).

| term | Estimate | SE | lower Cl | upper Cl | χ^2^ or z ^(1)^ | df | P |
| --- | --- | --- | --- | --- | --- | --- | --- |
| (Intercept) | -4,168 | 1,680 | -11,120 | -1,837 |  |  | ^(2)^ |
| Condition ^(3)^ | 0,338 | 0,750 | -1,628 | 2,402 | 0,195 | 1 | 0,659 |
| Apparatus ^(4)^ | 1,105 | 1,484 | -1,437 | 6,750 | 0,745 |  | 0,456 |
| Session ^(5)^ | -0,515 | 0,323 | -1,538 | 0,118 | 2,631 | 1 | 0,105 |
| Trial ^(6)^ | -0,797 | 0,431 | -2,059 | -0,150 | 6,107 | 1 | 0,013 |

^(1)^ Indicated are χ^2^ values with the exception of the test for apparatus for which we used a Wald test because the respective reduced model did not converge.

^(2)^ not indicated because of having a very limited interpretation

^(3)^ dummy coded with no partner being the reference category.

^(4)^ dummy coded with ball being the reference category.

^(5)^ z-transformed to a mean of zero and a standard deviation (sd) of one; mean and sd of the original variable were, 6.531 and 3.414, respectively.

^(6)^ z-transformed to a mean of zero and a standard deviation (sd) of one; mean and sd of the original variable were, 3.500 and 1.710, respectively.

**S9 Table** Object preferences: Detailed results of the Wilcoxon signed ranks tests.

Ball-Triangle: Z = 0.40, p = 0.69

Ball-Stick: Z = -3.29, p = 0.001

Ball-Square: Z = 0.25, p = 0.80

Triangle-Square: Z = -0.40, p = 0.69

Triangle-Stick: Z = -1.81, p = 0.071

Square-Stick: Z = -1.41, p = 0.159


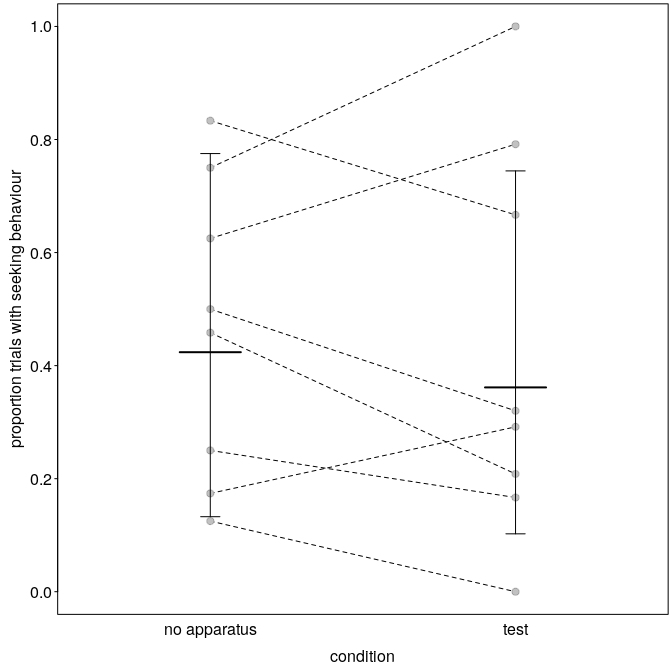
 **S2 Figure** Proportion of trials seeking, separately for each actor and condition. Dotted lines connect observations from the same individual. The thick horizontal lines with error bars indicate the fitted model and its confidence limits (for session and trial number centered to a mean of zero).

**S10 Table** Results of the model with seeking behaviour occurrence (no/yes) as the response (estimate, together with standard error, confidence limits, test results, as well as minimum and maximum of estimates after deleting sessions and actors one at a time).

| term | Est. | SE | lower Cl | upper Cl | χ^2^ | df | P | min | max |
| --- | --- | --- | --- | --- | --- | --- | --- | --- | --- |
| intercept | -0.307 | 0.768 | -1.877 | 1.237 |  |  | ^(1)^ | -0.725 | 0.075 |
| condtest^(2)^ | -0.262 | 0.534 | -1.374 | 0.814 | 0.221 | 1 | 0.638 | -0.687 | -0.045 |
| session nr.^(3)^ | 0.319 | 0.410 | -0.487 | 1.252 | 0.588 | 1 | 0.443 | -0.115 | 0.559 |
| trial nr.^(4)^ | -0.175 | 0.139 | -0.512 | 0.125 | 1.412 | 1 | 0.235 | -0.222 | -0.122 |

^(1)^ not indicated because of having a very limited interpretation

^(2)^ dummy coded with no apparatus being the reference category

^(3)^ z-transformed to a mean of zero and a standard deviation (sd) of one; mean and sd of the original variable were, 6.497 and 3.464, respectively

^(4)^ z-transformed to a mean of zero and a standard deviation (sd) of one; mean and sd of the original variable were, 3.487 and 1.710, respectively

**S11 Table** Results of the random effects part of the model with the occurrence of seeking behaviour as the response.

| grouping factor | effect^(1)^ | sd |
| --- | --- | --- |
| sessionID | trial nr. | <0.001 |
| sessionID | intercept | 1.658 |
| Actor | trial nr. | 0.000 |
| Actor | session nr. | 0.877 |
| Actor | cond.test | 0.298 |
| Actor | intercept | 1.896 |

^(1)^ intercept denotes a random intercepts effect, all others a random slope

#### **Begging behaviour and individual information on the animals**

**S12 Table** Number of trials in which the partner showed begging behavior in the test and no-apparatus condition (total number of trials per condition n=24).

| **Actor-Partner** | **Test-condition** | **No-apparatus condition** |
| --- | --- | --- |
| May-Dol | 24 | 18 |
| Fig-Fin | 19 | 15 |
| Fin-Mup | 7 | 4 |
| Dol-Hei | 0 | 3 |
| Kiw-Hei | 8 | 12 |
| Mon-Kiw | 5 | 11 |
| Mup-Mon | 4 | 6 |
| Zoz-May | 16 | 20 |

**S13 Table** The birds´ names, division in test group A and B, roles in the test, sex and age of the nine cockatoos. Note that Figaro participated only as actor, to prevent him from manufacturing his own stick tool from the wooden testing cage when being in the recipient-role. Heidi replaced him in the partner role with Dolittle as actor.

| **Group** | **Name** | **Role** | **Partner** | **Sex of the actor** | **Hatched** |
| --- | --- | --- | --- | --- | --- |
| A | Figaro | Actor | Fini | Male | 2007 |
|  | Mayday | Actor | Dolittle | Female | 2011 |
|  | Kiwi | Actor | Heidi | Male | 2010 |
|  | Muppet | Actor | Moneypenny | Male | 2010 |
| B | Fini | Actor | Muppet | Female | 2007 |
|  | Moneypenny | Actor | Kiwi | Female | 2010 |
|  | Dolittle | Actor | Heidi | Male | 2011 |
|  | Zozo | Actor | Mayday | Male | 2010 |
|  | Heidi | Partner | - | Female | 2010 |

1. **Test setup and apparatuses**


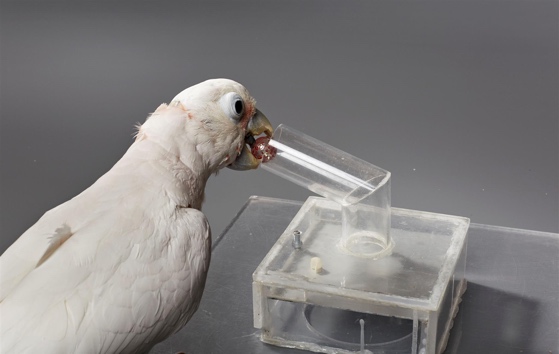

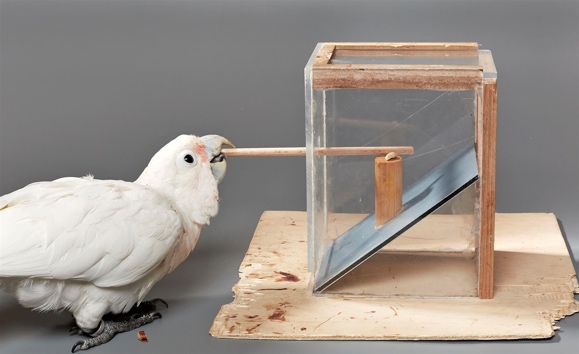


**S3 Figure** Left: ball-apparatus; Right: stick-apparatus. Only the respective tool is functional for each apparatus. The ball apparatus was made of a transparent plexiglass box that had a slanted tube attached on top, which prevented insertions of the stick tool. The reward rested on a platform within the box that was fixed with two magnets. By throwing the ball – a small marble – into the tube, the platform collapsed and the reward was released. The stick apparatus was made of transparent plexiglass box. The reward rested on a wooden platform and could be reached by inserting the stick tool and then poking off the reward. Pictures by Bene Croy.


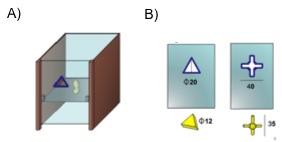


**S4 Figure** A) Shape-frame matching apparatus of the study by Habl & Auersperg (2017). The apparatus was made of wood with a plexiglass front. Similar to the ball-apparatus there was a collapsible platform inside held by a magnet. The reward rested on this platform and could be released through a slit at the bottom. B) The front wall could be exchanged and had either a triangle or a star shape cut into it.

**
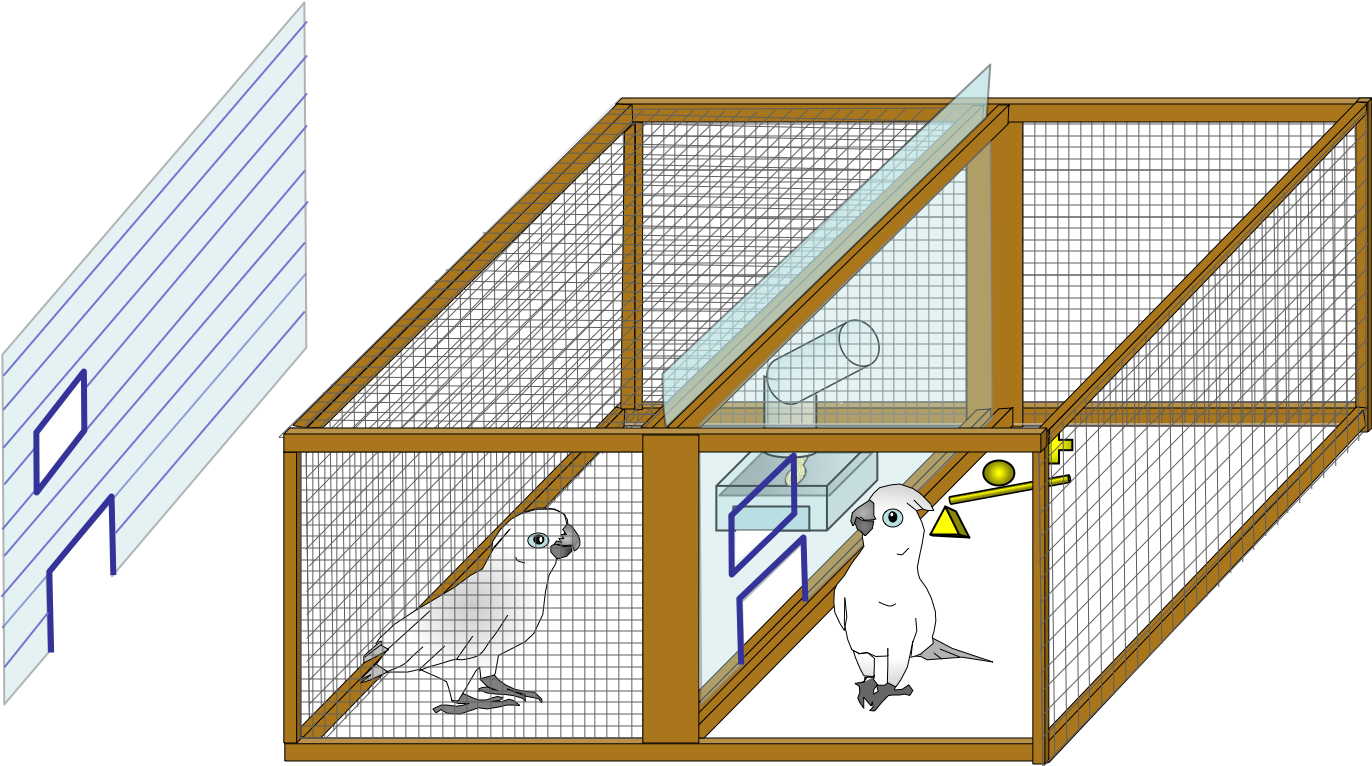
**

**S5 Figure** Birds were sitting next to each other in adjacent test compartments, separated by a plexiglass wall with two exchange windows (one on ground level, the other at eye-level). Tools were place in the actor´s compartment, whereas the partners side contained the baited apparatus, except in the no-apparatus condition. Left: In order to facilitate the visual recognition of the windows the plexiglass wall was marked with horizontal lines (2.4 cm apart) and the frame of the two exchange windows was colored. To ensure that the birds could not reach the apparatus in the partner´s compartment themselves, the testing apparatus (either ball- or stick-apparatus) was placed diagonally opposite of the exchange windows and screwed onto the table to prevent the partner bird from shaking it and thereby releasing the reward.

**
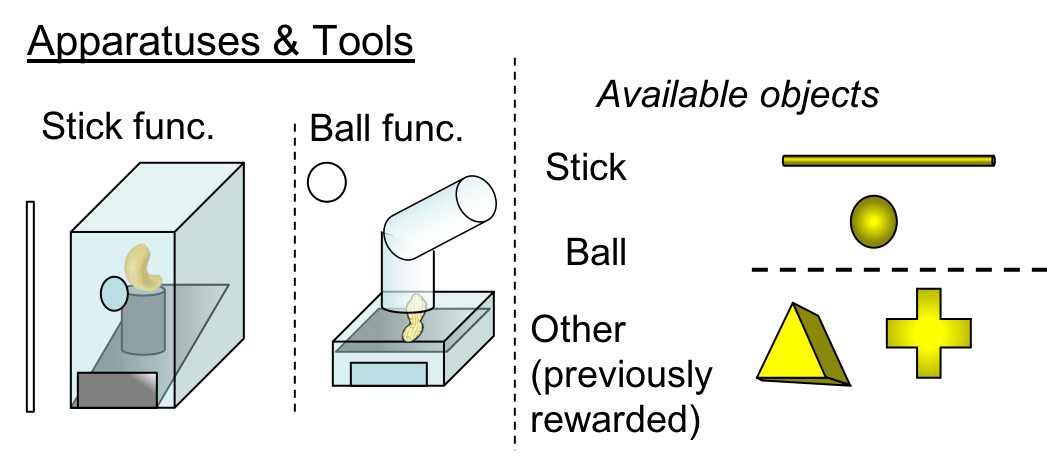
**

**S6 Figure** Stick and ball apparatus and available objects used in the test. Ball, stick and two distractor tools (associated with food in a previous study (Habl & Auersperg, 2017) but non-functional in present study) were placed in the actor´s compartment while, depending on the condition, none or one of the two apparatuses was placed in the recipient´s compartment.
